# Western Medical Schools

**Published:** 1844-01

**Authors:** 


### WESTERN MEDICAL SCHOOLS.

Kemper College, at Saint Louis, is said to have 90 students in attendance on the medical lectures, this winter. At Cincinnati there are reported to be about 180. A gentleman residing at Lexington, writes to a friend in this city, that the number at that school is 212. The number at the Louisville Medical Institute is 243. We have not heard from either the new school at St. Louis, or the New Orleans school.

